# Association between periodontitis and postoperative complications in hospital medical surgical procedures: a systematic review

**DOI:** 10.21142/2523-2754-1104-2023-177

**Published:** 2023-12-28

**Authors:** Alfredo Cueto Urbina, Javiera Guzmán Opazo, Katherine Sagredo Ramírez, Miguel Parra Parra, Silvia López De Blanc

**Affiliations:** 1 Public Health Division, School of Dentistry, University of Valparaiso, Chile. miguel.parra2@yahoo.com, kathsagredo.ramirez@gmail.com, jaavi.guzman@gmail.com, alfredocuetourbina@yahoo.es Public Health Division School of Dentistry University of Valparaiso Chile miguel.parra2@yahoo.com kathsagredo.ramirez@gmail.com jaavi.guzman@gmail.com alfredocuetourbina@yahoo.es; 2 Department of Oral Pathology and Stomatology, School of Dentistry, National University of Cordoba. Cordoba, Argentina. silvia.lopezdeblanc@unc.edu.ar Universidad Nacional de Córdoba Department of Oral Pathology and Stomatology School of Dentistry National University of Cordoba. Cordoba Argentina silvia.lopezdeblanc@unc.edu.ar

**Keywords:** periodontal diseases, periodontitis, postoperative complications, preoperative care, Surgery, enfermedades periodontales, periodontitis, complicaciones postoperatorias, cuidados preoperatorios, cirugía

## Abstract

**Background:**

Periodontitis is potentially harmful in the perioperative period due to biofilm generating a environment for bacteria to spread and colonize other anatomical areas, which can generate a potential risk of infection, delayed healing, increased morbidity, and even induce avulsion in intubated patients, and subsequent aspiration or ingestion of teeth with increased mobility.

**Objective:**

Associate the presence of periodontitis and postoperative complications in patients who underwent an in-hospital medical surgical procedure.

**Methods:**

A systematic review based on studies extracted from PubMed and Scopus was carried out on June 10, 2020, based on the Population, Intervention, Comparison and Result search strategy. As inclusion criteria, the studies had to include all the disaggregated terms of the research question, have a publication date of less than 15 years, and the target population had to have undergone elective hospital medical-surgical interventions. The exclusion criteria corresponded to not presenting an analytical or experimental observational study design, not having made a periodontal clinical diagnosis of the study subjects, and not expressing in the results the presence of postoperative medical-hospital complications. Articles were assessed for quality by supplementing the STROBE guideline and Newcastle Ottawa, for risk of bias by supplementing the STROBE guideline and the Cochrane Collaboration handbook tool.

**Results:**

A total of 131 articles were obtained, which were subjected to a selection process, resulting in 5 final analytical observational studies. A meta-analysis was performed and determined that periodontitis was a risk factor to postoperative complications after surgical procedures with an OR = 4,76; 95%CI [1,11-20,41].

**Conclusions:**

Optimize the guidelines for assessing quality and risk of bias can make their comparison with other studies complex, however it was determined in a statistically significant way that patients with periodontitis have a higher risk of generating postoperative complications after a medical hospital surgery.

## RESUMEN

**Introducción:**

La periodontitis es potencialmente dañina en el perioperatorio debido a que el biofilm genera un ambiente para que las bacterias se propaguen y colonicen otras áreas anatómicas, lo que puede generar un riesgo potencial de infección, retraso en la cicatrización, aumento de la morbilidad e incluso inducir avulsión en pacientes intubados, y aspiración o ingestión posterior de dientes con mayor movilidad.

**Objetivo:**

Asociar la presencia de periodontitis y complicaciones postoperatorias en pacientes sometidos a un procedimiento médico quirúrgico intrahospitalario.

**Métodos:**

Se realizó una revisión sistemática a partir de estudios extraídos de PubMed y Scopus el 10 de junio de 2020, basándose en la estrategia de búsqueda de Población, Intervención, Comparación y Resultados. Como criterios de inclusión, los estudios debían incluir todos los términos desagregados de la pregunta de investigación, tener una fecha de publicación menor a 15 años y la población objetivo debía haber sido sometida a intervenciones médico-quirúrgicas hospitalarias electivas. Los criterios de exclusión correspondieron a no presentar un diseño de estudio observacional analítico o experimental, no haber realizado un diagnóstico clínico periodontal de los sujetos de estudio y no expresar en los resultados la presencia de complicaciones médico-hospitalarias posoperatorias. Se evaluó la calidad de los artículos complementando la guía STROBE y Newcastle Ottawa, y el riesgo de sesgo complementando la guía STROBE y la herramienta del manual de Colaboración Cochrane.

**Resultados:**

Se obtuvo un total de 131 artículos, los cuales fueron sometidos a un proceso de selección, dando como resultado 5 estudios observacionales analíticos finales. Se realizó un metaanálisis y se determinó que la periodontitis fue un factor de riesgo de complicaciones posoperatorias después de procedimientos quirúrgicos con un OR = 4,76; IC95% [1,11-20,41].

**Conclusiones:**

Optimizar las guías para evaluar la calidad y el riesgo de sesgo puede hacer compleja su comparación con otros estudios, sin embargo, se determinó de manera estadísticamente significativa que los pacientes con periodontitis tienen mayor riesgo de generar complicaciones posoperatorias luego de una cirugía médico hospitalaria.

## INTRODUCTION

The presence of Periodontitis is potentially harmful in the perioperative period due to biofilm generating a favorable environment for bacteria to spread and colonize other anatomical areas^1^, which can generate a potential risk of infection, delayed healing, increased morbidity^2^, and even induce avulsion in intubated patients, and subsequent aspiration or ingestion of teeth with increased mobility^3^. In addition, it should be noted that the development of a postoperative complication implies an increase in the number of days of hospitalization and a more significant economic expense^2^.

Immunocompromised individuals, such as patients with comorbidities, do not easily eliminate bacteremia^4^, so periodontitis can influence systemic pathologies and vise versa^5^^-^^10^; poor oral health can influence the development of pulmonary infection, diabetes, arteriosclerotic disease, rheumatism, infective endocarditis, hypertension, cardiovascular disease, among others^11^^-^^18^. Therefore, these patients should undergo a preoperative dental evaluation.

Currently, in Chile, there are various clinical guidelines^19^^-^^22^, such as the Clinical Guide for total hip endoprosthesis in persons over 65 years of age, which indicates that once osteoarthritis has been confirmed, patients should undergo a preoperative dental evaluation and treatment to eliminate septic foci of oral origin^20^. Likewise, the Clinical Guide for preventing oral mucositis in people with cancer^21^ indicates that before starting oncological treatment, the patient should receive oral treatment with a therapeutic and preventive approach to reduce the risk of complications associated with the execution of this treatment. Similarly, other guides^19^^-^^22^ indicate that patients should undergo a preoperative dental evaluation, but there is no established protocol or requirement for dental discharge for most elective medical-surgical hospital surgeries^23^.

It has been reported that periodontal pathogens and their products can circulate in the bloodstream through associated bacteremia, thereby invading endothelial cells and promoting systemic vascular inflammation^24^^-^^26^. In this respect, several studies indicate that periodontal treatment can positively modify inflammatory markers, C-Reactive Protein, fibrinogen, and leukocyte count^17^. Therefore, adequate oral and dental health during the preoperative period can prevent postoperative complications.

Multiple studies have demonstrated that adequate preoperative oral health can prevent postoperative complications such as pneumonia and surgical site infection after cancer surgery^27^^,^^28^. Therefore, the question that arises is: Is the presence of pre-existing periodontitis preceding a medical-surgical procedure associated with the development or not of postoperative complications?

If the association is confirmed, it would become necessary to protocolize in more detail the approach to oral treatment prior to any hospital medical-surgical procedure, not only those already addressed in the health system; thus requiring the dentist to be actively and regularly involved in the pre-surgical phase to assess the need for dental treatments as an intervention measure to minimize additional costs and optimize patient care.

The purpose of this research was to update whether there is an association between the presence of periodontitis and postoperative complications in patients undergoing an in-hospital medical surgical procedure.

Given the aforementioned, it was hypothesized that the presence of pre-existing periodontitis preceding a hospital medical-surgical procedure is associated with the development of postoperative complications.

## MATERIALS AND METHODS

The present study consisted of a systematic review, following the guidelines of the PRISMA statement^29^ and the Cochrane manual for systematic reviews^30^.

Regarding the eligibility criteria for the search, it was based on the principles addressed in the research question employing the Population, Intervention, Comparison and Outcome search strategy (PICO), where the population corresponded to patients undergoing medical-hospital surgical interventions; the intervention was the presence of Periodontitis; the control to those without the presence of Periodontitis; and the outcome corresponding to the incidence of postoperative complications. Subsequently, these were adapted to Mesh or free-text terms (keywords) that yielded the highest number of articles.

Based on the above, the definitive terms for the search key were obtained:

• P="Risk factors", “Surgery”,
• I ="Periodontal diseases",
• C="Dental Care",
• O="Postoperative", "Complications" y "Postoperative complications".

The Boolean operators OR, AND, and NOT were included.

The databases used for the search were MEDLINE and Scopus; full access to the studies was obtained through an electronic server (PROXY) provided by the University of Valparaíso. The search in Scopus required indicating the search field by selecting the ALL option and including the term "Postoperative complications". The complete search key was then obtained:

- Pubmed: (((((((surgery)) OR ("risk factors") AND (periodontal diseases)) AND ("dental care")) AND (postoperative)) AND (complications)) NOT ("dental implants")) NOT ("periodontal surgery")) NOT ("third molar").

- SCOPUS: ALL ( surgery ) OR ALL ( "risk factors" ) AND ALL ( "periodontal diseases" ) AND ALL ( "dental Care" ) AND ALL ( postoperative ) AND ALL ( complications ) OR ALL ( "postoperative complications" ) AND NOT ALL ( "dental implants" ) AND NOT ALL ( "periodontal surgery" ) AND NOT ALL ( "third molar" ).

Subsequently, inclusion and exclusion criteria were applied. The inclusion criteria included all the broken-down terms of the research question, publication date less than 15 years, and the target population had to have undergone elective hospital medical-surgical interventions. Conversely, the exclusion criteria corresponded to not presenting an analytical or experimental observational study design, not having made a clinical periodontal diagnosis of the study subjects, and not expressing in their results the presence of postoperative medical-hospital complications.

Two investigators performed the search in parallel, while a third reviewer identified differences in case of controversy in the results to obtain and confirm data. No filtering was conducted concerning the type of article or language due to the poor results obtained when applying them. Both searches were conducted simultaneously on June 10, 2020. One article was previously selected on May 26, 2020, in Pubmed, thus being classified as a manual search article.

Duplicates were eliminated from the search performed. Then titles were reviewed, resulting in the removal of some articles due to them not being related to the research question; finally, the exclusion criteria were applied.

The synthesis of results was primarily based on the application of absolute frequencies concerning the number of healthy patients or patients with Periodontitis and postoperative complications with respect to the total sample. In addition, measures of association such as Odds ratio, relative risk, and p-value were used to determine the association between Periodontitis and postoperative medical-hospital complications.

The risk of bias assessment was established based on the table published by the Cochrane Handbook for experimental studies^31^ modified to assess the most recurrent systematic errors in analytical observational studies. In addition, the STROBE guideline^31^ criteria were used to inform on the design and methods of the study, and these were distributed into domains as recommended by the Cochrane manual^30^. Finally, each bias was broken down into its component biases, and the latter was associated with six domains. 

The three most frequent biases of analytical observational studies (selection, reporting, and confounding) were broken down into six domains^32^^,^^33^. 

The importance of the domains varied based on the type of study, as there were biases that were more relevant to assess depending on this^32^^,^^33^.

For case-control and retrospective cohort studies, the key domains were: methods of participant selection and sample homogenization, and for prospective cohort studies: incomplete outcome data. The key domains for all studies were: exposure and outcome measurement methods, confounder control methods, and selective reporting of results.

Each article was classified as low, high risk of bias, or unclear risk of bias assessment depending on whether it met the key domains requested.

In the individual bias assessment of each study, "low risk of bias" was defined as low risk of bias for all key domains; "unclear risk of bias" was defined as unclear for one or more key domains; and "high risk of bias" was defined as unclear for one or more key domains. In turn, concerning the analysis of risk between studies, it was defined as "low risk of bias" when most of the information comes from studies with low risk of bias; "unclear risk of bias" when most of the information comes from studies with low or unclear risk of bias; and "high risk of bias" when the information coming from studies with a high risk of bias is sufficient to affect the interpretation of the results.

Each article was analyzed individually, and the level of risk of bias was defined as low or high risk, determined unanimously among the three reviewers. For studies with a low risk of bias, most of the information comes from studies with a low risk of bias; therefore, it is plausible and unlikely to alter the results. On the other hand, for high-risk studies, it is considered that the proportion of information coming from studies with a high risk of bias is sufficient to affect the interpretation of results, which weakens their confidence. 

All the studies were observational, so the quality analysis was based on the STROBE statement^31^ with certain modifications. For example, items 12d, 13b, and 16c were not considered, and item 16c was not taken into account in cases the investigators did not declare it. The analysis was further complemented with the criteria established by the Newcastle Ottawa scale^34^, which is based on three main dimensions: selection, comparability, and results/exposure of interest, where critical points were established that respond to these dimensions, these correspond to 4, 6a, 7, 8, 12a, 14a, 16a, 15, 13a and 20. The studies had to meet a minimum percentage of 60% from this evaluation and include the critical points mentioned above. All the studies underwent this process, where they were examined by two reviewers, while the third reviewer identified differences in case of controversies, finally reaching a consensus. 

Subsequently, a pooled risk calculation (meta-analysis) was carried out using R project software version 4.0.0. By means of a random effect analysis determining the heterogeneity of the studies present. A Forest plot was performed to determine the point estimate and its respective 95% confidence interval. Also, a Funnel plot was performed in order to identify publication bias. 

## RESULTS

**Figure 1:**
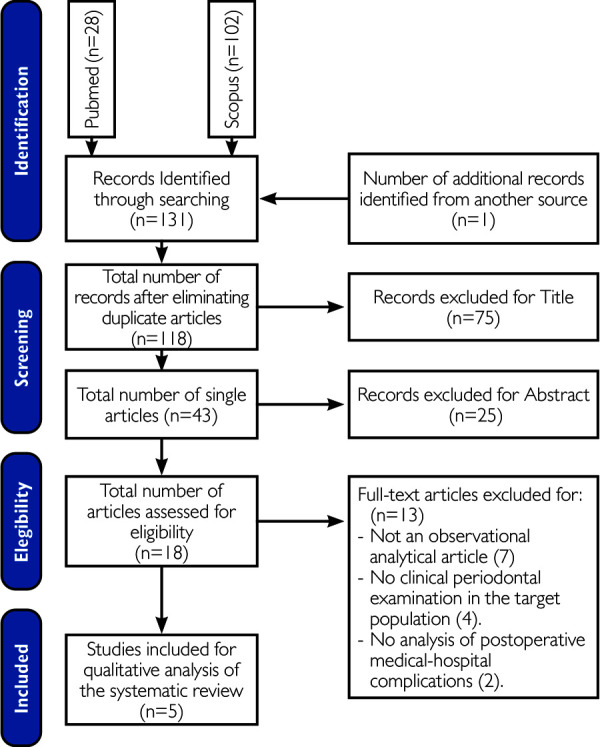
Flowchart of study selection

A total of 131 articles were retrieved from the search (Figure 1), of which 28 came from Pubmed, 102 from SCOPUS, and 1 obtained manually. After eliminating duplicates, 118 were left, then 75 studies were removed based on the title, leaving 43 in total. Then, 25 were removed as their abstract was not related to the research question; finally, the exclusion criteria were applied, obtaining a total of 5 final articles.

Among the characteristics of the latter (Table 1), it should be noted that they were conducted in university hospitals, except for Nakamura^35^, which was carried out in a general hospital. The years of publication ranged from 2009 to 2019, with a sample of both men and women, with an age range of 51 to 75 years. All surgical procedures performed were elective surgeries of different fields.

**Table 1:** Characteristics of the selected articles. The main postoperative complications are reported, although they are not the only ones.

Authors			Nakamura et al. ^35^	Nishikawa et al. ^36^	Mirzashahi et al. ^37^		Bágyi et al. ^38^	Sato et al. ^39^
Year of publication			2011	2019	2018		2009	2016
Study Design			Prospective cohort	Retrospective cohort	Case-Control		Prospective cohort	Retrospective cohort
Source of the institution			General Hospital	University Hospital	University Hospital		University Hospital	University Hospital
Mean Age (years)			65 ±10	Median 68 IQR: [61-75]	57 ± 6		70 ± 6	CG: 65 ±8 IG: 65 ± 8
Sample			209	341	78		23	529
Sex		Masculine	131	208	19		11	451
		Femenine	78	133	59		12	78
Surgical Procedure			Heart valve surgery	Gastrointestinal surgery	Elective spinal surgery		Brain surgery	Esophagectomy
Follow-up period			60 ±16 (months)	Median 3,6 IQR: [0,84 - 6,77] (years)	1 (year)		2 (days)	Undetermined
Validated diagnostic instrument			No	Clavien-Dindo^40^	Center for Disease Control definition of SSI^41^		American Thoracic Society Guidelines^42^	Clavien-Dindo^40^
Postoperative complications			Prosthetic valve endocarditis	Postoperative infectious complications	SSI		Postoperative pneumonia	Postoperative pneumonia

IQR: inter quadrant range, GC: Control group, IG: Intervention group, SSI: Surgical site infection.

The study by Nishikawa^36^, published in 2019, corresponded to a retrospective cohort study with a sample of 341 individuals who underwent gastrointestinal surgery. The medical history recorded was: body mass index, smoking, arterial hypertension, stroke, rheumatoid arthritis, acute myocardial infarction, hemodialysis, and corticosteroid use. The average follow-up period was 3.63 years.

The study by Mirzashahi^37^ published in 2018 was a case-control study with a sample of 78 individuals undergoing elective spinal surgery, with a 1-year follow-up. The medical history recorded was body mass index, history of spinal surgery, smoking, diabetes mellitus, drug use, and comorbidities.

The study by Bágyi^38^, published in 2009, was a prospective cohort study with a sample size of 23 individuals undergoing brain surgery and a follow-up period of 2 days. The medical antecedents were preoperative chest x-ray and laboratory tests. 

The study by Sato^39^, published in 2016, was a retrospective cohort study with a total sample of 529 people undergoing esophagectomy. The medical history reported was nutritional status and respiratory function, although the follow-up period was not reported.

The study by Nakamura^35^, published in 2011, was a prospective cohort study with a sample size of 209 individuals undergoing heart valve surgery with a follow-up of 60 ± 16 months. Medical history included smoking, diabetes mellitus, valvular disease, and other cardiac diseases. 

For the evaluation of postoperative complications (Table 1), 4 of the articles used validated instruments^40^^-^^42^, except for Nakamura et al. ^35^. Furthermore, the latter was the only study in which the number of patients with Periodontitis and postoperative complications was zero, which is why no association was established. Some postoperative complications described were surgical site infection, pneumonia, deep organic space infection, anastomotic leak, urinary tract infection, and prosthetic vascular endocarditis.

Table 2 describes the periodontal diagnosis achieved in the studies. Each of them used a different diagnostic method.

**Table 2:** Description of periodontal diagnosis and postoperative complications.

Authors	Nakamura et al. ^35^	Nishikawa et al. ^36^	Mirzashahi et al. ^37^	Bágyi et al. ^38^	Sato et al. ^39^
Standardization of periodontal diagnosis	Based on clinical experience	Six sites were examined per tooth^43^	No	Examinations performed by a single professional	Examinations performed by a single professional
Periodontal diagnostic scale	Based on the need for tooth extraction Yes/ No	According PPD: - Mild: <4 (mm) - Moderate: 4-6 (mm) - Severe: ≥ 6 (mm) Plaque index	Gingivitis, tooth mobility or gingival recession Yes/No	Scoring system according to severity and gravity	Probing depth
Periodontal classification according to severity	- None - Mild - Severe	- Mild - Moderate - Severe - Edentulous	No	- Mild - Moderate - Severe	- None - Mild - Severe - Edentulous
Number of patients according to diagnosis or periodontal damage	- Severe Periodontitis: 104 - Control (none/mild): 105	- Periodontitis: 298 - Healthy: 43	- Periodontitis: 37 - Healthy: 41	Undetermined	Intervention Group: - Periodontitis treated: 160 - Healthy: 67 Control Group: - Untreated periodontitis: 297
Distribution of patients with periodontitis according to severity	Periodontitis - None or mild:105 - Severe:104	Periodontitis - Mild: 36 - Moderate:180 - Severe:82	No	Periodontal score - High:7 - Low:16	Periodontitis - Mild:91 - Severe:69
Number of patients with postoperative complications	3 (100%)	48 (100%)	8 (100%)	5 (100%)	69 (100%)
Number of patients with periodontitis and postoperative complications	0 (0%)	36 (75%)	6 (75%)	5 (100%)	49 (71%)
Association	No	Yes	Yes	Yes	Yes
Measure of association	No information	Odds ratio: 2.09 CI [95%]: 1.045-4.183 P-value: 0.037	Odds ratio: 3.77 CI 95% [0.711-19.985] P-value: 0.049	Relative risk: 3.5 CI [95%]: 1.09-11.29 P-value: <0.0001	Relative risk: 2.52 CI [95%]: 1.38-4.78 P-value: 0.0025

PPD: probing pocket depth CI: Confidence interval

Nishikawa et al. ^(^^36^ classified Periodontitis according to probing depth into mild, moderate, severe, and edentulous. Meanwhile, Mirzashahi et al. ^(^^37^ classified active periodontal disease based on the presence of gingival inflammation, tooth mobility, and gingival recession. However, Sato et al. ^(^^39^ only limited themselves to diagnosing according to probing depth without naming the parameters used.

Nishikawa et al. ^(^^36^ determined that periodontal disease was an independent risk factor for infectious postoperative complications (p=0.037). Additionally, Mirzashahi et al. ^(^^37^ determined that it correlated significantly with surgical site infection (p=0.049). Bágyi et al. ^(^^38^ established that the number and severity of coexisting periodontal diseases were significantly higher in patients with postoperative pneumonia compared to the control group (p= <0.0001), finally Yusuke Sato et al. ^(^^39^ reported that patients who did not have preoperative dental treatment were at higher risk of having postoperative pneumonia than the group that did have treatment (p=0.002).

**Figure 2:**
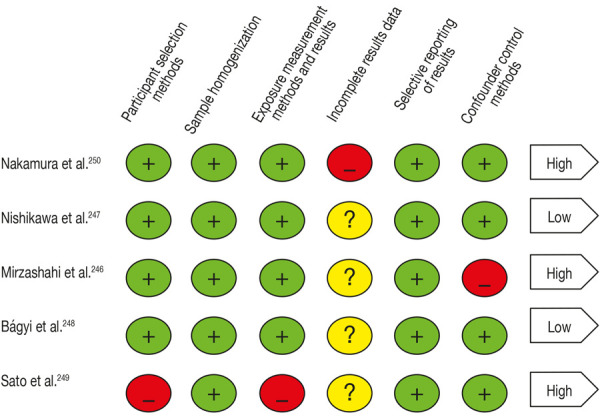
Risk of bias assessment. Adapted from the Cochrane Collaboration tool^30^

Regarding the results of the risk of bias assessment (Figure 2), the low-risk studies identified were: Nishikawa et al. ^(^^36^ and Bágyi et al. ^(^^38^. Conversely, most showed a high risk of bias: Nakamura et al. ^(^^35^ for presenting incomplete outcome data, Mirzashahi^37^ for having shortcomings in the methods to control confounding factors, and finally Sato et al. ^(^^39^ for the methods in the selection of participants and the homogenization of the sample. Overall, the interpretation of the risk of bias among the studies was of high risk, meaning that most of the information came from studies with a high risk of bias, which was sufficient to affect the interpretation of the results.

According to the quality assessment tool, derived from an adaptation of the aforementioned STROBE statement^31^ (Table 3). The article by Nishikawa et al. ^(^^36^^)^ included all the critical points, being also the study with the highest quality standard with 77.4%, followed by Bágyi et al. ^(^^38^ with 74%, Nakamura et al. ^(^^35^ with 70.9%, Sato et al. ^(^^39^ with 66.6% and finally, Mirzashahi^37^ with 63.3%. These last two authors complied with 8 of 10 critical points. Thus, there was concordance between the STROBE guideline^31^ and the points established from the Newcastle-Ottawa scale^34^.

**Table 3:** Quality assessment of the selected studies. Critical items boxes marked with gray.

STROBE^31^ Score	Nakamura et al. ^35^	Nishikawa et al.^36^	Mirzashahi et al.^37^	Bágyi et al.^38^	Sato et al.^39^
1a	NO	YES	YES	YES	YES
1b	YES	YES	YES	YES	YES
2	YES	YES	YES	YES	YES
3	YES	YES	YES	YES	YES
4	YES	YES	YES	YES	YES
5	YES	YES	YES	YES	YES
6a	YES	YES	YES	YES	NO
6b	NO	NO	NO	NO	NO
7	YES	YES	YES	YES	YES
8	YES	YES	YES	YES	NO
9	YES	YES	NO	YES	YES
10	NO	NO	NO	NO	NO
11	YES	YES	YES	YES	YES
12ª	YES	YES	NO	YES	YES
12b	YES	YES	YES	YES	YES
12c	NO	NO	NO	NO	NO
12d	N/A	N/A	N/A	N/A	N/A
12e	NO	NO	NO	NO	NO
13a	YES	YES	YES	YES	YES
13b	N/A	N/A	N/A	N/A	N/A
13c	NO	NO	NO	NO	NO
14a	YES	YES	YES	YES	YES
14b	NO	NO	NO	NO	NO
14c	YES	YES	N/A	YES	N/A
15	YES	YES	YES	YES	YES
16a	YES	YES	NO	YES	NO
16b	YES	YES	N/A	NO	N/A
16c	N/A	N/A	N/A	N/A	N/A
17	YES	NO	YES	YES	YES
18	YES	YES	YES	YES	YES
19	YES	YES	YES	YES	YES
20	YES	YES	YES	YES	YES
21	YES	YES	YES	YES	YES
22	NO	YES	YES	YES	YES

N/A: not applicable.

**Figure 3:**
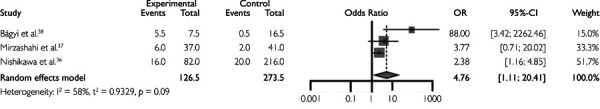
Combined risk of the association between postoperative complications and periodontitis

A pooled risk calculation was performed to identify the association between postoperative complications and Periodontitis, resulting in an OR of 4.76 and CI of [1.1; 20.41], as shown in Figure 3.

## DISCUSSION

In this review of observational studies, a positive and statistically significant association was established between Periodontitis and postoperative complications, independent of the method of data collection and analysis.

Regarding the periodontal diagnosis achieved in the articles, different systems were used. Only Nishikawa et al. ^(^^36^ relied on a validated instrument, which corresponded to the 2015 Clinical Practice Guideline for Periodontal Treatment of the Japanese Society of Periodontology^43^, contrastingly, the other authors used their own diagnostic criteria. In this regard, Bágyi et al. ^(^^38^ made a diagnosis according to severity and seriousness, classifying it as dental calculus, gingivitis, mild Periodontitis, moderate and severe Periodontitis. 

In their study, Nakamura et al. ^(^^35^ compared a group with severe Periodontitis (considered according to the need for tooth extraction and curettage of the lesion) with another group composed of patients with no Periodontitis and mild Periodontitis, which compromised the quality of the control group. Therefore, in the future, it is essential to use the same method to diagnose Periodontitis in order to compare studies.

The classification of postoperative complications differed among studies. Nevertheless, a universal classification has been sought for decades. One attempt is due to the Clavien-Dindo classification^40^, and more recently, Strasberg, who in 2009 proposed the Accordion system^44^. Even so, the Clavien-Dindo system has been widely used, with an exponential increase in recent years^40^. These classification systems for the severity of complications are based on the treatment that these complications require^45^. Furthermore, it is the one the authors of this review suggest continuing using in the future.

In terms of follow-up periods, there was no standardized length of time as shown in the studies; in this regard, one of them covered a period of 2 days^38^, which made comparability difficult.

As shown in Table 3, all of them complied with the STROBE guidelines31 with the minimum percentage of sufficient quality, but not all the points considered "critical". Moreover, according to Figure 2 of the bias assessment, 3 of the 5 articles were considered high risk; hence the results could be less reliable and valid^35^^,^^37^^,^^39^. Consequently, further research is required to ensure high levels of evidence.

It is sometimes controversial to recommend preoperative oral treatment, which consists of dental treatment for eliminating infectious foci immediately before surgery^46^, seen as it may have a paradoxical result. Unfortunately, few studies have a high level of evidence to clarify this situation^47^. 

The retrospective study by Nakamura et al. ^(^^35^ was the only one that found no relationship between periodontal disease and postsurgical complications, mainly because its objective was to establish the optimal timing of periodontal treatment. The study evaluated tooth extractions according to periodontal diagnosis in patients undergoing heart valve surgery, and the complication sought was mainly prosthetic valvular endocarditis, a low-frequency condition, of the order of 1%. No other significant morbidity was present in the sample. In addition, there was no control group of patients with Periodontitis that would not receive dental treatment, in this case, dental extraction. 

As periodontal microbiota is packed in the gingival sulcus, it disrupts the epithelial integrity by inflammation. The intimate contact between the ulcerated epithelium and the bloodstream^48^ generates the diffusion of bacterial products such as endotoxins, lipopolysaccharides, and inflammatory response of the host itself to the rest of the body^49^. Considering the effects mentioned above and considering that the associated bacteremia generates altered inflammatory and immunological responses^50^, postoperative complications are probably associated with this pathophysiological mechanism^51^. 

Another mechanism of interest occurs during the intubation time: when artificial ventilation is used, it generates aspiration of pathogens that colonize the oral cavity into the lungs and make it difficult to maintain oral hygiene, which increases the risk of postoperative pneumonia^52^^-^^54^.

In Japan, it was concluded that lack of professional preoperative oral care might increase the risk of postoperative complications^45^ and that periodontal disease might compromise the surgical outcome, increase morbidity, increase the need for additional therapies, delay the healing process and extend hospitalization^2^. In this regard, the US Center for Disease Control and Prevention (CDC) recommends treating infections remote to the surgical site prior to operations^41^^,^^55^.

According to a systematic review of patients undergoing cardiovascular surgery, all patients who developed postoperative complications had either Periodontitis or a high-risk periodontal diagnosis^56^. Likewise, the American Heart Association and the American College of Cardiology advise performing a dental evaluation before cardiac surgery^16^.

The instruments used in the present review for the assessment of quality^31^^,^^34^ and risk of bias^30^^,^^31^ correspond to widely validated and used tools, which were adapted and modified for use in a justified manner to optimize the analysis of the results obtained. However, this could have generated the limitation of making their comparability with other studies difficult.

It is recommended to further evaluate alternatives for dental management prior to in-hospital medical-surgical procedures.

Periodontal disease was consistently identified as an independent risk factor for infectious postoperative complications, as has been proposed by some authors of the studies analyzed.

Pre-existent Periodontitis preceding a hospital medical-surgical procedure is related to infection of the anatomical site, pneumonia, anastomotic leakage, urinary tract infection, among other complications. Furthermore, patients without any periodontal treatment before the surgeries had a higher risk of developing postoperative pneumonia than the treated group.

In view of this, it is necessary to have a multidisciplinary and preventive approach to evaluate the oral health of patients in a preoperative period in order to achieve timely treatment and effectively reduce the risk of postoperative complications, which in turn, would have a positive impact in economic and health terms. To this end, the development of clinical guidelines and protocols to be applied transversally before hospital surgeries should be encouraged, where the dentist plays an essential role as a general and oral health professional.
